# Screening and identification of genes associated with cell proliferation in cholangiocarcinoma

**DOI:** 10.18632/aging.102766

**Published:** 2020-02-10

**Authors:** Li Guo, Yaodong Zhang, Zibo Yin, Yaya Ji, Guowei Yang, Bowen Qian, Sunjing Li, Jun Wang, Tingming Liang, Changxian Li, Xiangcheng Li

**Affiliations:** 1 Department of Bioinformatics, Smart Health Big Data Analysis and Location Services Engineering Lab of Jiangsu Province, School of Geographic and Biologic Information, Nanjing University of Posts and Telecommunications, Nanjing 210023, China; 2 Hepatobiliary Center, The First Affiliated Hospital of Nanjing Medical University, Nanjing 210029, China; 3 Jiangsu Key Laboratory for Molecular and Medical Biotechnology, School of Life Science, Nanjing Normal University, Nanjing 210023, China

**Keywords:** cholangiocarcinoma (CCA), cell proliferation, synthetic lethality, therapeutic target

## Abstract

Cholangiocarcinoma (CCA), an aggressive tumor with poor prognosis, is a malignant cancer with increasing incidence and mortality rates. It is important to survey crucial genes in CCA to find and design potential drug targets, especially for those genes associated with cell proliferation that is a key biological process in tumorgenesis. Herein, we surveyed genes associated with cell proliferation via a comprehensive pan-cancer analysis. Candidate genes were further analyzed using multiple approaches, including cross-analysis from diverse molecular levels, examination of potential function and interactions, and additional experimental validation. We primarily screened 15 potential genes based on 11 validated genes, and these 26 genes were further examined to delineate their biological functions and potential roles in cancer treatment. Several of them were involved synthetically lethal genetic interactions, especially for *RECQL4*, *TOP2A*, *MKI67* and *ASPM*, indicating their potential roles in drug design and cancer treatment. Further experimental validation indicated that some genes were significantly upregulated in several cancer cell lines, implying their important roles in tumorigenesis. Our study identifies some genes associated with cell proliferation, which may be potential future targets in molecular targeted therapy.

## INTRODUCTION

Cholangiocarcinoma (CCA) is a highly fatal cancer, with often challenging disease diagnosis, treatment, and prognosis [1]. Most CCA patients with advanced disease have short median survival times of less than 1 year after diagnosis [2]. The molecular mechanism underlying the malignant progression of CCA remains ambiguous. Very limited clinical treatment options are available because CCA is insensitive to conventional chemotherapy and radiation treatment [3]. Therefore, how to develop novel therapeutic strategies for CAA is crucial and urgent, especially for recently concerned strategy of molecular targeted therapy.

Over the past decade, abnormally expressed genes associated with CAA have been studied. These genes have been shown to largely contribute to CCA occurrence and development. Some of these genes are related to the processes of cell growth, DNA mismatch repair, immortalization, the cell cycle, and other biological pathways [3]. Specifically, *p53* and *p16INK4A* contribute to oncogenesis in the biliary tract, and are often abnormally expressed in cancer [4, 5]. For cancer cells with unlimited proliferation, some genes are crucial for the cell proliferation process, which is coordinated with cell death during normal cell development. Genes associated with cell proliferation can be used as markers to track the cell state. Abnormal expression of such genes, especially up-regulation patterns, may indicate the deregulation of cell proliferation. These genes may also provide potential therapeutic targets for targeted therapy strategies. Therefore, the screening and identification of abnormally expressed genes during CCA would provide potential molecular targets for exploiting novel chemopreventive and therapeutic strategies.

Herein, we systematically analyzed genes associated with cell proliferation based on abnormal expression profiles through a pan-cancer analysis with multiple validations. First, we identified a series of experimentally validated genes associated with cell proliferation in the published literatures. Candidate genes were identified based on their deregulation in CCA in combination with the pan-cancer analysis. We then performed functional and drug response analyses, examined synthetic lethal genetic interactions, and evaluated other levels, to understand the potential biological roles of these candidate genes. Specific candidate genes were further validated using protein profiling and experimental validation to validate their expression patterns. This study provides information about genes associated with cell proliferation in CCA, which may be used as potential drug targets in therapeutic strategies.

## RESULTS

### Overview of expression landscape in cholangiocarcinoma

**Figure 1:**
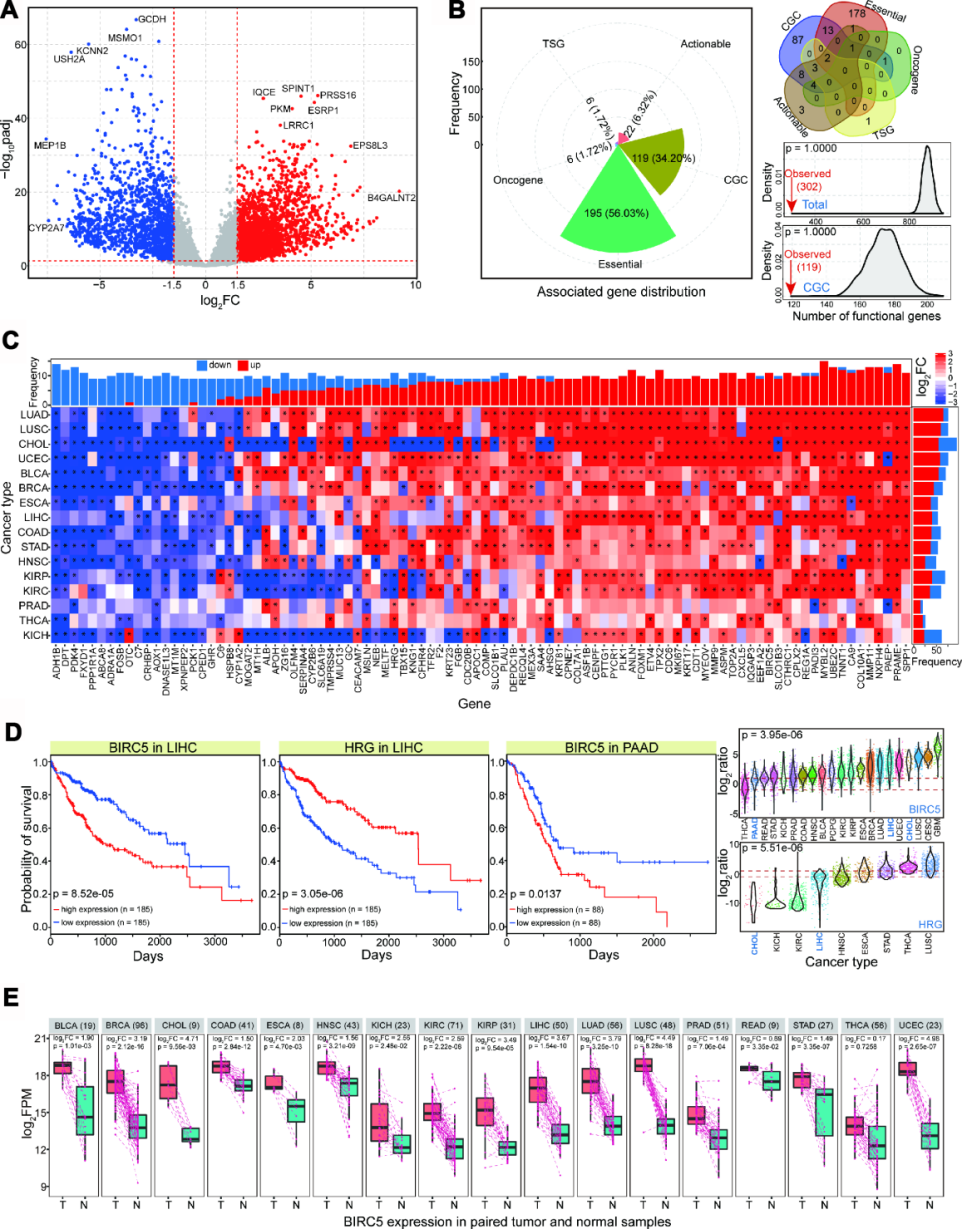
**Abnormally expressed genes in CCA (named CHOL in TCGA) and their expression patterns across diverse cancer types.** (**A**) Gene expression profiles in CHOL. Red point shows up-regulated gene (log_2_FC > 1.5 and padj < 0.05), blue point shows down-regulated gene (log_2_FC < -1.5 and padj < 0.05), and grey point shows normally expressed gene. Some abnormally expressed genes are presented their names. Red dotted lines show thresholds of log_2_FC (1.5 and -1.5) and padj (0.05). The baseMean values in DeSeq of these involved genes are not less than 50. (**B**) For screened abnormal genes, distributions of associated functional genes (mainly including essential gene, CGC, oncogene, etc) are presented. Compared with random result in the equal number of normally expressed genes, fewer abnormal genes are identified as functional genes. (**C**) Pan-cancer analysis of expression distributions of 94 screened dominant abnormal genes in CHOL (baseMean value > 500, log_2_FC > 2 or < -2, padj < 0.05), and all of these genes are abnormally expressed (log_2_FC > 2 or < -2, padj < 0.05) at least in 9 cancer types. (**D**) Examples of survival analysis of *BIRC5* and *HRG* in LIHC and PAAD, and their expression distributions across different cancer types. (**E**) *BIRC5* expression in paired tumor and normal samples, and the log_2_FC value and p value are also presented based on paired t-test. T indicates tumor samples, and N indicates paired normal samples. The total sample size is presented after cancer name.

We first analyzed the CCA expression landscape to understand the differential expression profiles. Using this approach we identified many deregulated genes with significantly upregulated or downregulated expression patterns (Figure 1A and Supplementary Figure 1A). Abnormal expression patterns are partly caused by negatively regulating by non-coding RNAs, especially for microRNAs (miRNAs). For example, miR-21 may be oncogenic by inhibiting *PDCD4* and *TIMP3* in CCA [6], and miR-204 and miR-320 can regulate *Bcl-2* and *Mcl-1*, respectively [7]. miRNA:mRNA interactions are widespread based on validated datasets in Starbase [8] (Supplementary Figure 1B). Some pairs show significant negative correlations, implicating their interactions and potential regulatory roles of miRNAs. Indeed, many miRNAs have been shown to have important regulatory roles and to contribute to abnormal mRNA expression profiles [9–11].

Our screening of abnormally expressed genes revealed different enriched KEGG (Kyoto Encyclopedia of Genes and Genomes) pathways for upregulated and downregulated genes (more enriched pathways were found in downregulated genes), indicating potentially different biological functions between these deregulated genes (Supplementary Figure 1C). Few of the abnormally expressed genes were identified as functional genes, such as core essential genes, oncogenes, and cancer gene census (CGC) (Figure 1B and Supplementary Figure 1D). Random testing showed that functional genes were more likely to be normally expressed than they were to be deregulated (based on 1,000 times, p = 1.0000, Figure 1B). These results suggest that the crucial nature of functional genes means that they are strictly regulated and stably expressed owing to their important biological roles. Hallmarks of cancer could be detected, such as growth signal self-sufficiency and insensitivity to antigrowth signals (Supplementary Figure 1E), implying that these abnormally expressed genes have biological roles in tumorigenesis. To understand the dominant expression landscape in CCA, we first screened 94 abundantly expressed deregulated genes (|log_2_FC| > 2 and padj < 0.05). These genes were abnormally expressed at least in nine cancer types, and the majority showed consistent expression distribution across various tissues (Figure 1C). Consistent abnormal expression patterns across diverse cancer types indicates that these genes may contribute to basic biological processes, particularly in relation to their potential roles in tumorigenesis.

Our CCA sample sizes were insufficient to perform prognostic analysis. Therefore, to investigate the potential relationship between abnormally expressed genes and prognosis, we selected some genes and performed survival analysis in cancer types related to CCA. These mainly included liver hepatocellular carcinoma (LIHC) and pancreatic adenocarcinoma (PAAD). The molecular pathogenesis of CCA is similar to that of hepatocellular cancer, and some dominant risk factors are associated with both cancer types. Moreover, transcriptome analysis has confirmed some common genomic features between the two cancer types [12–14]. These deregulated genes, including *BIRC5* and *HRG* (histidine-rich glycoprotein), may be correlated with prognosis, although they show dynamic expression across diverse tissues (Figure 1D). *BIRC5* was upregulated in intrahepatic cholangiocarcinoma, and may contribute to the development of diagnostic and therapeutic strategies [15]. Another gene, *HRG*, was significantly deregulated in intrahepatic cholangiocarcinoma and may inhibit tumor growth and metastasis [16]. Furthermore, to validate the expression patterns of *BIRC5* and *HRG* across individuals, paired samples were used to present their expression patterns. The results of paired analysis indicated that the expression of these genes was relatively stable (Figure 1E and Supplementary Figure 1F), while significant expression differences were observed across diverse cancer types. Based on current sample distributions, these results further validate that differential analysis produces stable results.

### Screening of genes associated with cell proliferation

*MKI67*, a typical proliferation marker, is a key gene in cell proliferation [17–19], and it is used to screen candidate cell proliferation related genes. To increase our screening sensitivity, we included another 10 genes associated with cell proliferation in breast cancer (*BIRC5*, *CCNB1*, *CDC20*, *CEP55*, *NDC80*, *TYMS*, *NUF2*, *UBE2C*, *PTTG1*, and *RRM2*) [20]. Together, these 11 validated genes were used to further screen potential cell proliferation associated genes.

**Figure 2:**
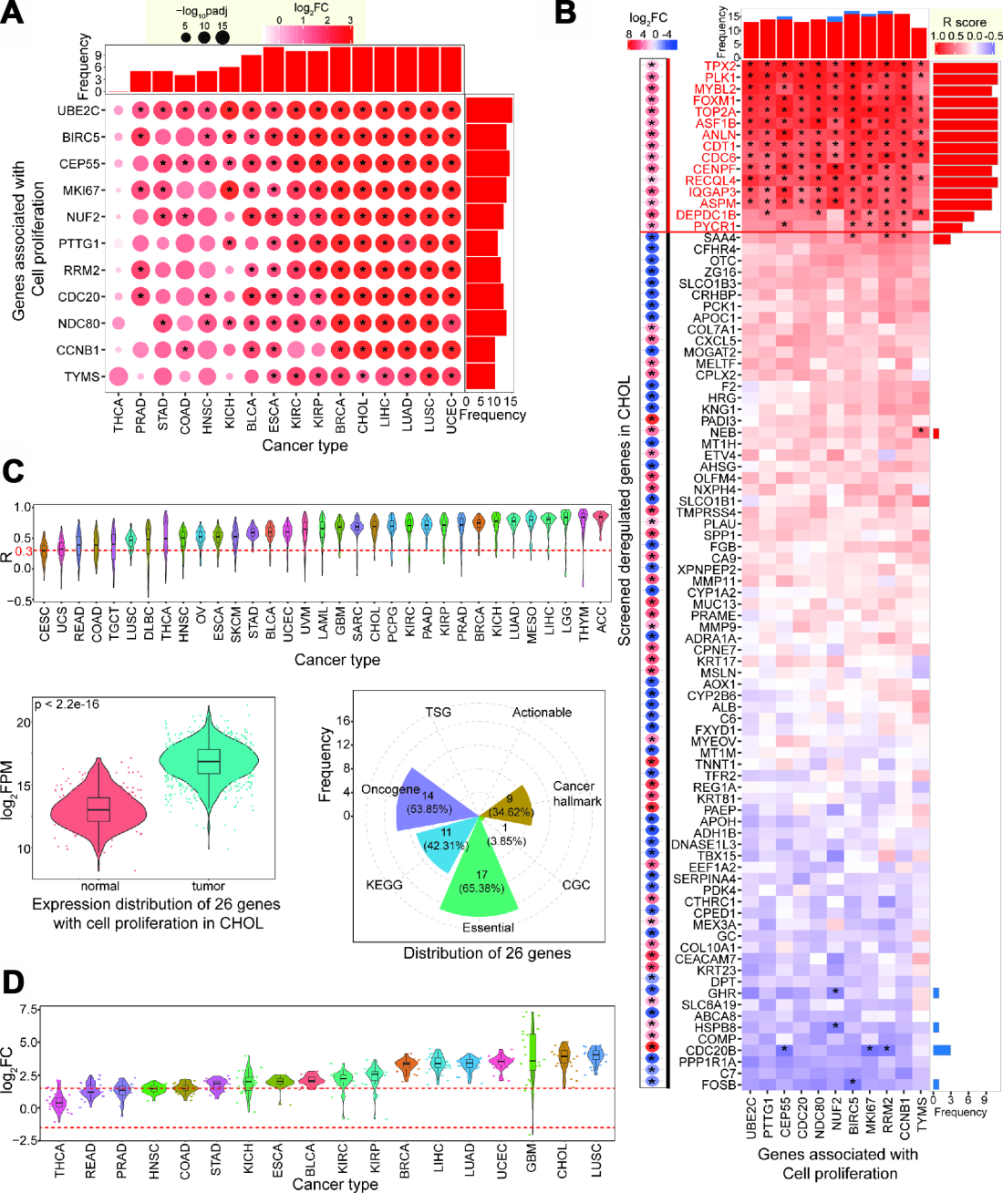
**Screening candidate genes associated with cell proliferation.** (**A**) Expression patterns of validated 11 genes associated with cell proliferation across diverse cancer types. (**B**) Correlation analysis of screened abnormally expressed genes with 11 validated genes in CCA (repeated genes have been removed from 94 genes). Deregulated patterns are also presented on the left. * indicates significant statistical result (padj < 0.05 for the left figure or FDR < 0.05 for the right figure, and these involved genes show up-regulated or down-regulated expression with |log_2_FC| > 2 and padj < 0.05). Fifteen up-regulated genes with positive correlation with more than 4 validated genes are believed as candidate genes with cell proliferation. (**C**) Distribution of correlations of 15 candidate genes across different cancer types, showing many of them have positive correlations with validated genes. The total 26 genes show significant difference between normal and tumor samples in CCA (p < 2.2e-16). (**D**) These 26 genes show dynamic expression patterns across various cancer types, but most are up-regulated.

These 11 genes showed significantly upregulated expression patterns in CCA, and most were also upregulated in other cancer types (Figure 2A). Upregulated expression of these genes supports the contention that they may have crucial cellular roles and contribute to the occurrence and development of diverse cancer types. The observed consistent expression trends indicate that these genes have common features in tumorigenesis. Based on the 94 deregulated genes identified by screening (Figure 1C), we performed a comprehensive correlation analysis in CCA. To filter potential genes associated with cell proliferation, we screened this data set for genes with expression that significantly positively correlated with the 11 validated genes. Using this approach, we identified 15 genes, all of which were significantly upregulated in CCA, and were positively correlated with *MKI67* and at least three other validated genes (R > 0.30, FDR < 0.05) (Figure 2B). To understand the expression patterns of these 15 genes in diverse cancer types, we analyzed their R coefficient distributions with 11 validated genes. We found that these screened 15 genes tended to show positive R coefficient distributions across diverse tissues (Figure 2C), implying that they may be crucial in pathophysiological cancer processes via over-expression. Therefore, we collected 26 genes associated with cell proliferation. The 11 validated genes were previously reported in other cancer types, so we analyzed their expression in CCA to predict their potential roles in tumorigenesis. All 26 genes were significantly upregulated in tumor samples (p < 2.2e-16, Figure 2C). Similar results were also observed across diverse cancer types (Supplementary Figure 2A).

We found that these 26 genes showed dynamic expression across different tissues, but consistent over-expression patterns were observed (Figure 2D). The stable over-expression patterns in diverse cancers implies that these genes are crucial for the occurrence and/or development of cancers. Some of the identified genes have important roles in tumorigenesis. For example, *FOXM1* and *MYBL2* may be key regulators of cell proliferation in non-small lung cancer [21], *MYBL2* is associated with cell proliferation in liver cancer cells [22], *TPX2* may be a potential therapeutic target in genomically unstable cancer cells [23], and *PLK1* may contribute to autophagy in osteosarcoma cells [24].

### Function analysis showing important biological roles for 26 genes

**Figure 3:**
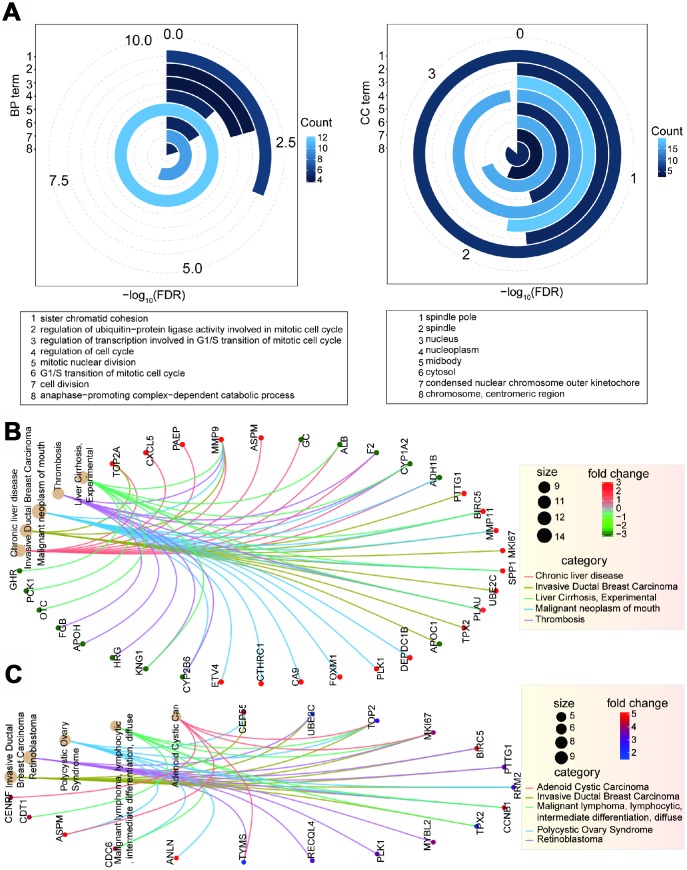
**Function analysis of deregulated genes.** (**A**) Enriched significant GO terms based on 26 candidate genes using David platform. (**B**) Enriched disease network of 94 deregulated genes.(**C**) Enriched disease network of screened 26 candidate genes.

To understand how the collected 26 genes correlate with cancer, we examined whether they have crucial cellular roles. We found that most of these genes have important roles or contribute to biological functions. Of the 26 genes, 17 (65.38%) were identified as core essential genes, 14 (53.85%) were oncogenes, 11 (42.31%) were enriched in KEGG pathways, and nine (34.62%) were correlated with hallmarks of cancer (Figure 2C). Several genes, including *CCNB1*, *CDC20*, *CDC6*, *PLK1*, and *PTTG1*, had multiple roles as core essential genes, oncogenes, and roles as hallmarks of cancer and in KEGG pathways. The cell cycle was the most enriched KEGG pathway (FDR = 0.0150), while no significant pathway was enriched in the 94 deregulated genes identified through screening (without 11 validated genes). Gene Ontology (GO) analysis for biological process terms revealed that only platelet degranulation was enriched in the 94 deregulated genes. However, eight terms were significantly enriched in the 26 genes associated with cell proliferation (Figure 3A). Taken together, these results imply that the 26 genes identified are crucial and have important roles in multiple biological processes.

The cell cycle and oocyte meiosis were the most dominantly enriched pathways for these 26 genes, and growth signal self-sufficiency and insensitivity to antigrowth signals were the most enriched cancer hallmarks (Supplementary Figure 2B). Some candidate genes showed significant over-expression patterns and were crucial nodes in the cell cycle process. These indicated that the screened 26 genes have important biological roles, especially in relevant pathways or biological processes associated with cell proliferation (Supplementary Figure 2B and 2C). Similar results were also obtained for cellular component terms (Figure 3A and Supplementary Figure 3A), indicating that these 26 genes have important biological roles and contribute to multiple crucial biological pathways.

To understand the complex association between these screened genes and other disease types, we predicted the potential associations of gene sets and diseases. Using the 94 genes deregulated in CCA, we found that several disease types were enriched. These include malignant neoplasm of mouth, invasive ductal breast carcinoma, chronic liver disease, thrombosis, and liver cirrhosis (Figure 3B). Some diseases were also enriched for the 26 identified genes, including invasive ductal breast carcinoma, retinoblastoma, and polycystic ovary disease (Figure 3C, Supplementary Figure 2C and 3A). Although there were 15 common genes in both of these gene sets, only invasive ductal breast carcinoma was the common enriched disease type. We further queried the roles the filtered 26 genes in multiple biological processes (Supplementary Figure 2C and 3A, 3B). Some of these genes were crucial nodes in the cell cycle pathway, could be enriched in several significant GO terms, and some were related with other disease types. Taken together, these results provide evidence for their roles in basic biological and pathophysiological processes, especially in tumorigenesis, and support their potential in targeted cancer therapy.

### Potential synthetic lethal genetic interactions for 26 genes

Synthetic lethality, a type of genetic interaction, is emerging as a promising potential anticancer strategy that may be used to identify new antibiotic or therapeutic targets [25, 26]. For the 26 genes with potential roles in cell proliferation, it is necessary to further query potential negative genetic interactions between them and other genes. Such information will contribute to identifying their potential roles in anti-cancer strategy.

**Figure 4:**
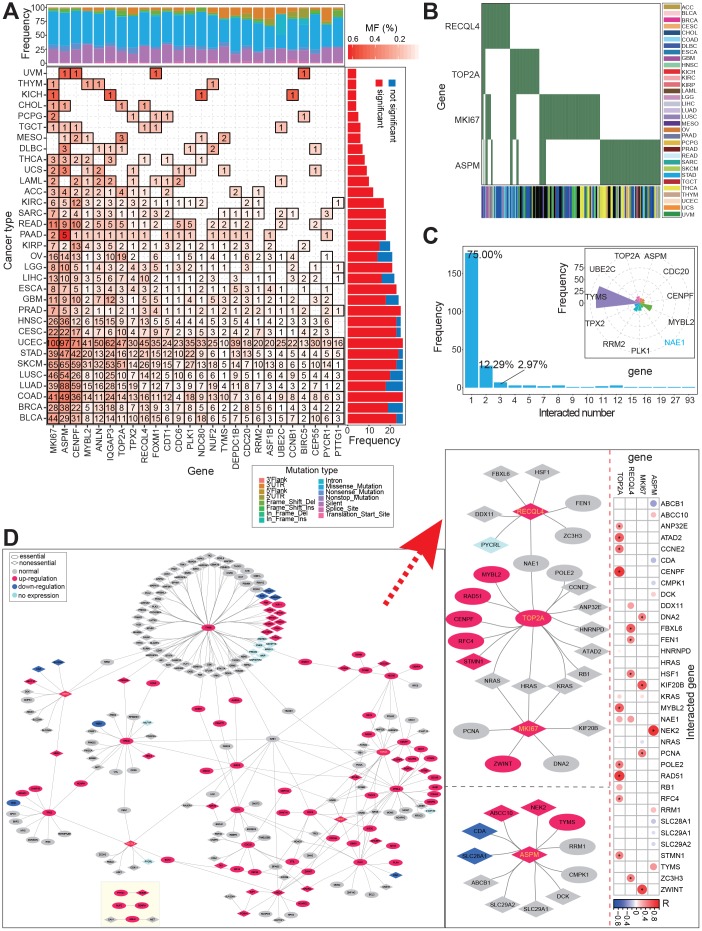
**Potential synthetic lethal genetic interactions in CCA.** (**A**) Distribution of mutation genes across cancer types based on 26 screened genes. (**B**) Distribution of mutation gens across individuals for 4 genes with mutation. (**C**) Distribution of interacted numbers based on validated/predicted synthetic lethal genetic interactions, and several genes with more than 10 interactions are also presented. (**D**) Interaction networks among synthetic lethal genetic interactions based on 26 genes. Networks of 4 genes with mutations are detailed presented on the right, and the correlations with the 4 genes are also presented. * indicates significant correlations between these interacted genes (FDR < 0.05).

We analyzed the mutation profile of these screened genes across 33 cancer types, and identified mutated sites in many cancers (Figure 4A and 4B). In eight cancer types, mutations in all 26 genes were detected in patients. However, in patients with CCA, mutations were identified in only four genes: *MKI67*, *ASPM*, *TOP2A*, and *RECQL4* (Figure 4B). This may be a function of the small CCA sample size in this study. Mutation profiles in other cancer types, with larger sample sizes, were sufficient to show the mutation trends of these genes and their potential association with the occurrence of cancer (Figure 4B and Figure 4B).

Then, according to currently gene pairs with synthetic lethality (experimentally validated and predicted), these 26 genes were surveyed for potential gene pairs (pairs contained one candidate gene were collected). Negative interactions were detected between 23 candidate genes and another 213 genes, and a total of 284 gene pairs were finally filtered. Among of these, 75% of genes were detected in one interaction, and only 11 genes were detected in more than 10 interactions with other genes (with the exception of *NAE1*, all other genes were those screened and identified in our study). In particular, 93 interactions were detected for *TYMS* (Figure 4C). *TYMS* has been reported as an important gene in diverse cancers, including red blood cell folate and homocysteine concentrations [27], and metastatic colorectal cancer [28–30]. Further, the interaction network showed that these genes showed diverse expression patterns in CCA, and that the pattern of over-expression was dominant (Figure 4D). Four genes with mutations identified in patients with CCA, *RECQL4*, *TOP2A*, *MKI67*, and *ASPM*, were found to have complex interactions with other genes, some of which showed significantly positive expression correlations (Figure 4D). These potential gene interactions indicate that complex gene networks can be used to identify novel antibiotic or therapeutic targets, and that the interactions will be more complicated if non-coding RNAs are involved.

Moreover, among these 26 genes, only eight gene pairs, containing 12 genes, were detected. Nine of these 12 genes were identified as essential genes, and two of them were involved in mutation (Supplementary Figure 3C). All of the gene pairs with synthetic lethality implied negative interactions, indicating that potential drug targets can be screened based on synthetic lethality. Indeed, increasing attention is being paid to synthetic owing to the potential application as a drug target in novel treatment method.

### Potential roles in cancer pathway activity and drug response

**Figure 5:**
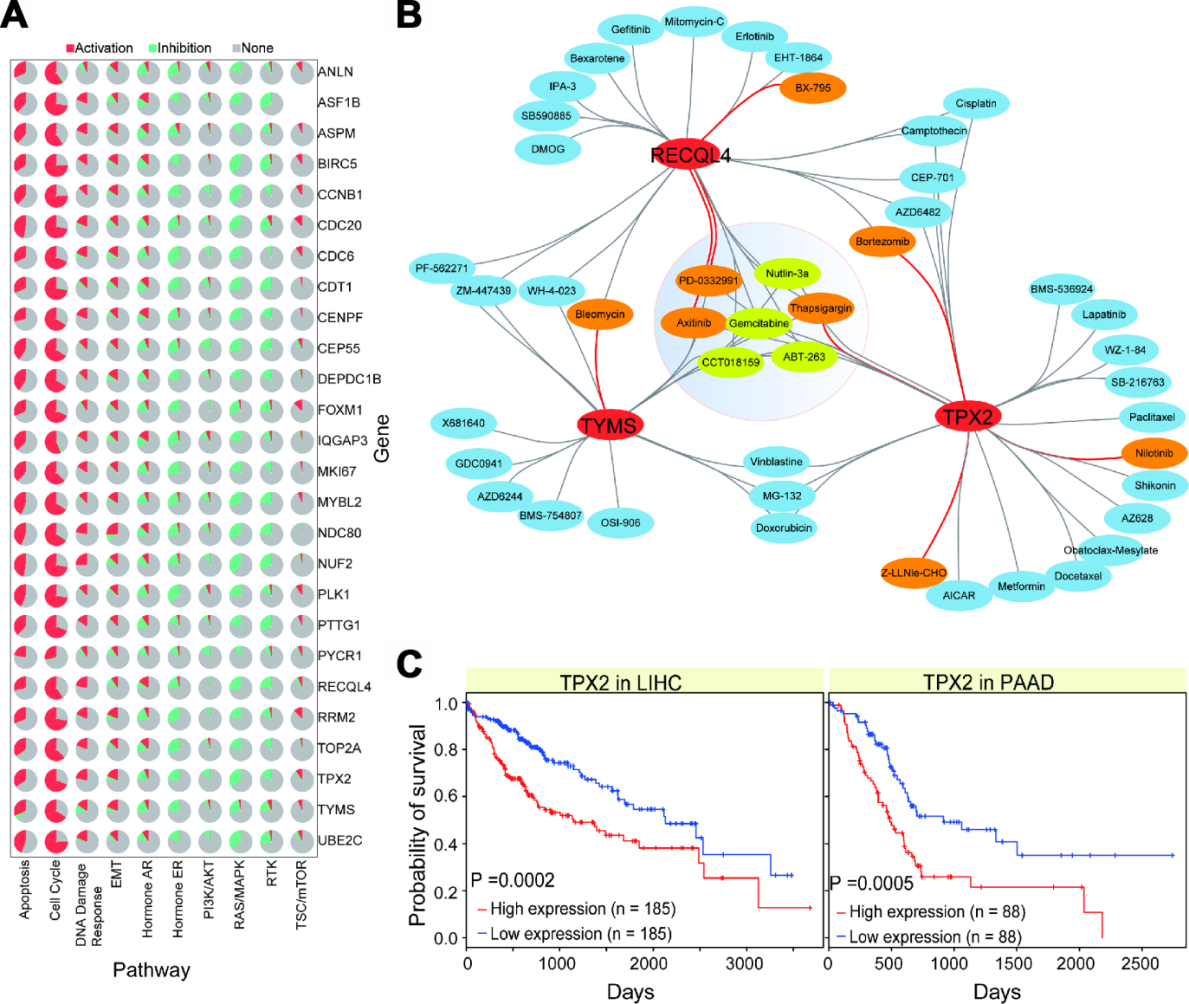
**Interactions of gene-drug and potential biological roles of some genes.** (**A**) Pie distributions of associations of 26 genes and pathways, and their roles in pathways, activation or inhibition, are also presented. (**B**) Gene-drug interactions in CCA. All of these involved drugs are predicted with positive (DR > 0.1, p < 0.05) or negative (DR < -0.1, p < 0.05) correlations. Red lines show significant positive or negative correlation. (**C**) Survival analysis of TPX2 in relevant cancer types showing their potential role in disease prognosis.

To understand the potential relevant cancer pathway roles of the 26 screened genes, we performed the relevant analysis using GSCALite [31]. This analysis allows us to predict activation or inhibition roles in cancer-related biological pathways. We found that most of these genes showed consistent roles in relevant pathways, including activation roles in cell cycle and apoptosis pathways, and inhibition roles in RAS/MAPK and hormone ER pathways (Figure 5A). These results indicate that these genes are crucial in diverse biological processes, and that their abnormal expression may lead to the activation or inhibition of normal biological pathways. Specifically, *CDC20* was associated with apoptosis (Supplementary Figure 3D), indicating that it contributes to the cellular apoptosis process. The functional role of *CDC20* has been reported in the cell cycle, proliferation, and apoptosis [32, 33], and it may present a potential novel cancer therapeutic strategy as a candidate target [34]. Our results indicate that these candidate genes are always abnormally enriched in cancer samples, and are prone to over-expression (Figure 2), implying a potential correlation between their abnormal expression and cancer processes.

Given the association between these genes and cancer, we analyzed their potential drug responses to understand their potential roles as drug targets. First, based on the 94 abnormally expressed genes in CCA, we found some of them showed significant correlations with drugs (Supplementary Figure 4A). For specific drugs, most related genes showed consistent gene-drug associations, indicating the existence of complex gene-drug interactions, and potential interactions between different genes (such as genetic interactions among genes). Then, for the screened 26 genes, only 3 of them had significant drug responses in CCA (Figure 5B and Supplementary Figure 4B). For example, *RECQL4*, *TYMS*, and *TPX2* showed correlations with some drugs, and multiple interactions were be detected between these genes. Although the CCA sample size obtained from The Cancer Genome Atlas (TCGA) was small, the significant correlations observed between genes and drugs validate that the identified genes of interest may be potential drug targets for the design of corresponding drugs. Further survival analysis indicated that most candidate genes were significantly correlated with prognosis in LIHC and PAAD, implying their potential roles in cancer prognosis (Figure 5C, Supplementary Figure 4C, and 4D). Some of them have been shown to have important roles in disease prognosis. For example, *TPX2* is associated with poor survival in gastric cancer [35], targeting *TPX2* can suppress tumor cell growth in prostate cancer, and *TPX2* is a potential therapeutic target and a prognostic indicator in clear cell renal cell carcinoma [36]. The roles of these candidate genes, their interactions with other molecules, and their potential roles as drug targets genes should be examined in future experiments.

### Protein expression profiling and experimental validation showing deregulated proteins

Functional analysis showed that some of genes of interest may be potential drug targets. To validate the expression levels of these relevant genes, we performed protein expression profiling using Triple TOF 5600in 11 paired CCA tissue samples.

**Figure 6:**
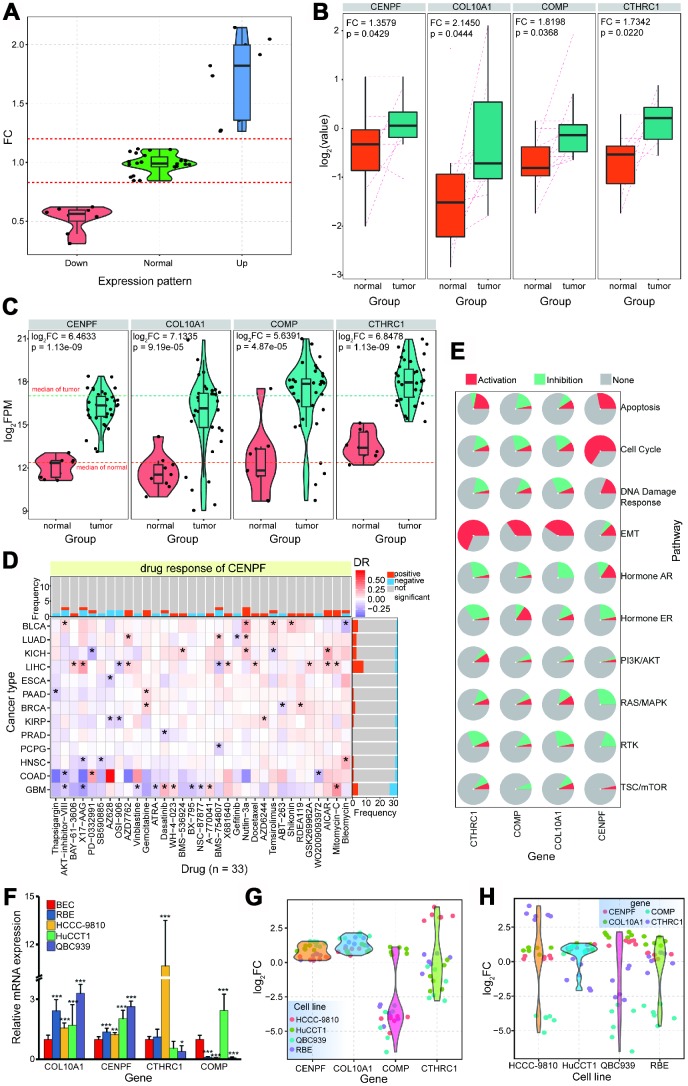
**Further expression patterns of relevant proteins via protein profiling.** (**A**) Expression distribution of detected proteins based on screened 94 genes. (**B**) The 4 proteins, *CENPF*, *COL10A1*, *COMP* and *CTHRC1*, are abnormally over-expressed in tumor samples (FC > 1.3, p < 0.05). (**C**) For the 4 up-regulated proteins, consistent expression patterns are detected in CCA at mRNA levels. (**D**) Drug responses for *CENPF* across different cancer types.* indicates significant correlations (p < 0.05). (**E**) Predicted roles of the 4 genes in biological pathways (activation or inhibition). (**F**). RT-PCR results for screened genes in different cell lines. * indicates p < 0.05, ** indicates p < 0.01, and *** indicates p < 0.001 (all experiments are repeated three times). (**G**) The detailed expression trends for each gene based on each experimental validation. (**H**) The detailed expression trends for each cell line based on each experimental validation.

First, the expression profiles of 94 abnormally expressed genes were queried, and 39 showed high enrichment levels (Figure 6A). Then, examination of the 26 upregulated genes revealed that four of them were identified as upregulated proteins that were consistent with mRNA expression result (Figure 6B and Figure 6C). Over-expression of these four genes at both mRNA and protein levels verified their roles in the carcinogenic process, and further drug analysis (based on diverse cancer types) verified that *CENPF* may be a potential drug target (Figure 6D). These four genes have important roles in biological pathways, including activation roles in EMT pathways, and *CENPF* has significant activation roles in apoptosis, cell cycle, and DNA damage response (Figure 6E and Supplementary Figure 4E).

Further experimental validation showed that both *COL10A1* and *CENPF* were significantly upregulated in four cell lines, and *CTHRC1* was significantly upregulated in the HCCC-9810 cell line specifically (Figure 6F–6H). Upregulation of *COMP* was validated via protein profiling, but *COMP* expression was inconsistent across the four cell lines used. *COMP* expression was significantly upregulated in CCT, but significantly downregulated in other cell lines (Figure 6G and 6H). Indeed, *CENPF* was mechanically linked to altered metabolism and progression in prostate cancer, and may be a crucial regulator [37]. Overexpression of *CENPF* may correlate with poor prognosis in breast cancer [38], *CENPF* may be a new prognostic biomarker in nonmuscle invasive bladder cancer [39], and the HnRNPR-CCNB1/CENPF axis contributes to gastric cancer proliferation and metastasis [40]. *FOXM1* and *CENPF* co-expression could be a potential robust prognostic indicator of poor survival and metastasis in prostate cancer [41], and these two genes may contribute to driving prostate cancer [42].

## DISCUSSION

Homeostasis of cell number is quite important for the maintenance of the architecture and function of normal tissues. However, cancer cells become masters of their own destinies by deregulating growth-promoting signals, and sustained proliferative signaling is a hallmark of cancer [43]. The detailed regulatory process in cancer cells is quite complex, and genes associated with cell proliferation may be crucial markers for tracking or assessing cell status. It is important to understand which of the genes that are crucial for cell proliferation are also crucial for abnormal cancer cells. This understanding will contribute to further screening and the identification of potential antibiotic or therapeutic drug targets.

We are particularly interested in genes associated with cell proliferation in CCA. Cell proliferation is an important process in tumorigenesis so we used a pan-cancer analysis to identify genes associated with cell proliferation. We performed a systematic analysis across different cancer types and tissues which allows for cross-validation. First, we screened for genes that were abnormally and dominantly expressed in CCA and further queried their expression patterns in other tissues. Genes with consistent expression levels were used to filter target genes. Validation of abnormally expressed genes in other tissues may provide additional gene expression pattern information and could implicate them as having important roles in biological processes. Second, 11 experimentally validated genes associated with cell proliferation were collected and their expression patterns were analyzed (*MKI67* is the most crucial gene, because it is a classical marker gene in cell proliferation). Candidate gene expression patterns were compared with those of the 11 validated genes and correlations were identified. Significantly positive expression correlation is crucial for screening genes associated with cell proliferation. As crucial components of the cell proliferation process, the expression levels of these genes may reflect cell proliferation. Third, candidate genes were further queried for their potential functions, which indicates their biological roles in the cell proliferation process. Using these methods, our primary screen identified 15 candidate genes, and further analysis revealed a total of 26 candidate genes. Most of these genes have important biological roles that have been previously reported, and functional analysis and validation revealed some of them as potential targets in cancer treatment, especially their roles in clinical anticancer therapies based on synthetic lethal genetic interaction.

Taken together, using a systematic analysis across different cancer types and tissues, we identified 26 candidate genes associated with cell proliferation. These genes may be crucial for the occurrence and development of CCA. Further validation should focus on their roles as potential therapeutic targets.

## MATERIALS AND METHODS

### Data source

Mutation data, RNA sequencing data, and clinical data for 33 cancer types were obtained from TCGA (https://tcga-data.nci.nih.gov/tcga/) using the “TCGAbiolinks” package [44] (http://doi.org/ 10.1093/nar/gkv1507). Other data used, including drug data, were obtained from public databases and/or published literatures, and they would be described in corresponding parts.

### Screening potential genes associated with cell proliferation

Differential expression profiles were estimated in diverse cancers using DESeq2 [45], and genes with abnormal expression profiles were filtered if |log_2_FC| > 1.5 and padj < 0.05 (adjusted p-values). Genes differentially expressed in CCA (named CHOL in TCGA database) were collected. To understand the roles of these genes in other cancer types, their expression patterns were queried in diverse tissues (abnormal expression profiles were collected from 16 cancer types, because some cancer type had small sample sizes in either the normal or tumor groups (< 8)). Genes involved in the cell proliferation process should have consistent features across different cancer types because they are important in the development of cancer. Therefore, it is important to assess the expression patterns of candidate genes in diverse cancer types, not only in CCA.

We then developed a panel of 11 genes with experimentally validated cell proliferation association. These included the *MKI67* cell proliferation genetic marker, and an additional 10 genes [20]. Potential aim genes were first screened based on their patterns of upregulation and their positive correlation with the 11 validated genes. Candidate genes, together with the 11 validated genes in other cancer type, were further analyzed to identify their biological roles.

### Function enrichment analysis

To understand the potential biological function of the screened mRNAs, we performed functional enrichment analysis using The Database for Annotation, Visualization and Integrated Discovery (DAVID) version 6.8 [46]. Simultaneously, to further understand how the potential candidate gene features related to their biological function in cancer physiology, we also analyzed potential distribution of these genes in related cancer hallmark [47] (http://software.broadinstitute.org/ gsea/msigdb/), CGC [48] (http://cancer.sanger.ac.uk/census), core essential genes (derived from the common essential genes from Hart et al. [49], Blomen et al. [50] and Wang et al. [51]), actionable genes [52], oncogene, and tumor suppressor gene [53] datasets.

To understand whether related genes were potential drug targets, genes were further queried for drug response using the Genomics of Drug Sensitivity in Cancer database (GDSC) [54]. Detailed drug response, including sensitivity or resistance, was mainly identified according to |DF| > 0.1 and p < 0.05.

Based on our pan-cancer analysis and the consistent expression features of important cell proliferation genes, we used clusterProfiler [55] to delineate the complex association between the genes involved and disease types.

### Estimating potential synthetic lethal genetic interactions

Recently, synthetic lethality, a genetic interaction first identified in yeast, has been widely considered as a means for identifying novel antibiotic or therapeutic targets [25, 26]. To examine synthetic lethality in the screened genes potentially associated with cell proliferation, we identified gene pairs with experimentally validated and predicted relevant interactions from the SynLethDB database [56], Syn-Lethality database [57], and published literature [58]. Interacting genes were completely screened based on the identified gene pairs, and expression patterns and gene characters were also queried for those genes with interactions to predict their roles as potential therapeutic targets.

### Survival analysis

To estimate the correlations of candidate genes with cancer prognosis, survival analysis was performed. The clinical data retrieved from TCGA included survival status, cancer stage and grade, survival time, and molecular subtype. The patients involved were divided into two groups according to the median expression level of a specific gene. We used a log-rank test to estimate the potential difference between the two groups. Statistical significance was assumed when the p value was less than 0.05.

### Randomization test

To determine the significance of a detected frequency of special gene classification, we performed a randomization test by randomly selecting normally expressed genes with equal numbers. This procedure was repeated 1,000 times (the significance was estimated based on the proportion of times), and was used to estimate whether the average correlation values observed were higher than the real average correlation.

### Hierarchical clustering analysis

We performed hierarchical clustering analysis of the most dominantly and abnormally expressed mRNAs in CCA to present their expression patterns across different cancer types using the R package “pheatmap” and the average distance algorithm. Simultaneously, correlation between genes and validated genes to be involved in cell proliferation were also assessed using similar methods.

### Protein profiling using Triple TOF 5600

To validate protein expression profiles in CCA, we collected 11 pairs of tumor and normal (adjacent-to-tumor samples) samples from 11 patients that were diagnosed with CCA in the First Affiliated Hospital of Nanjing Medical University, Nanjing, Jiangsu, China. To ensure sample homogeneity, all included patients were male with similar ages, and had similar disease processes. This study was conducted in accordance with the declaration of Helsinki and with approval from the Ethics Committee of Nanjing Medical University. Written informed consents were obtained from all participants.

Total protein (100 μg) was extracted from each sample solution and the protein was then digested with Trypsin Gold (Promega, Madison, WI, USA). Dried peptides were reconstituted in 0.5 M TEAB and processed using the 8-plex iTRAQ reagent (Applied Biosystems) following the manufacture’s protocol. Protein profiles from the paired samples were measured using the Triple TOF5600 System (AB SCIEX, Concord, ON) fitted with a Nanospray III source (AB SCIEX, Concord, ON) at LC-BIO Technologies (Hangzhou) CO., LTD. Differentially expressed proteins were identified using the paired Wilcoxon rank sum test, and only those with p < 0.05 and fold change > 1.2 were considered significant.

### Experimental validation using quantitative real-time RT-PCR

To validate the expression of the screened genes, particularly those that were overexpressed and verified by protein profiling, we selected four CCA cell lines (RBE, HCCC-9810, HuCCT1, and QBC939) to verify expression patterns based on BEC normal cell lines.

Total RNA was extracted from CCA cells using the TRIzol reagent (Invitrogen, Carlsbad, CA, USA). cDNA was generated from total RNA using a reverse transcription kit (Takara, Dalian, China). Gene expression was measured by qPCR (Lightcycler96, Roche, Basel, Switzerland) using a SYBR green kit (Yeasen, Shanghai, China) and following the manufacturer’s instructions. The used primers were shown in Table 1, and *36B4* was used as an internal control for normalization.

**Table 1:** Primers used for the real-time PCR.

**Gene**	**Forward Primer**	**Reverse Primer**
CENPF	5'-TTGTAAAGAAAGGGTTTGC-3'	5'- CCAGCTGTTGGTTTGGAGG -3'
COL10A1	5'-CTTCACTTGAATGGGAGGCACAAGG-3'	5'-TGCAAGGTGCTTTCATCAATGAACC-3'
COMP	5'-AACACGGTCACGGATGACGACTATG-3'	5'-CACAGAGCGTTCCGCAGCTGTTC-3'
CTHRC1	5'-TCATCGCACTTCTTCTGTGGA-3'	5'-GCCAACCCAGATAGCAACATC-3'

### Statistical analysis and network visualization

All statistical analyses were performed using R programming language (version 3.4.3), and included the Wilcoxon rank-sum test and trend test. Interactions between related genes, and drug-gene interactions were first estimated, and further network visualization was performed using Cytoscape 3.6.0 [59]. Venn distributions were generated using a publicly available tool (http://bioinformatics.psb.ugent.be/webtools/Venn/).

## Supplementary Material

Supplementary Figures
