# Supplementary material for: Screening and identification of genes associated with cell proliferation in cholangiocarcinoma
Source: Aging (Albany NY). 2020 Feb 10;12(3):2626–46. doi: 10.18632/aging.102766 (PMC7041743; doi:10.18632/aging.102766)
Supplement: Supplementary Figures [file aging-12-102766-s001..pdf]

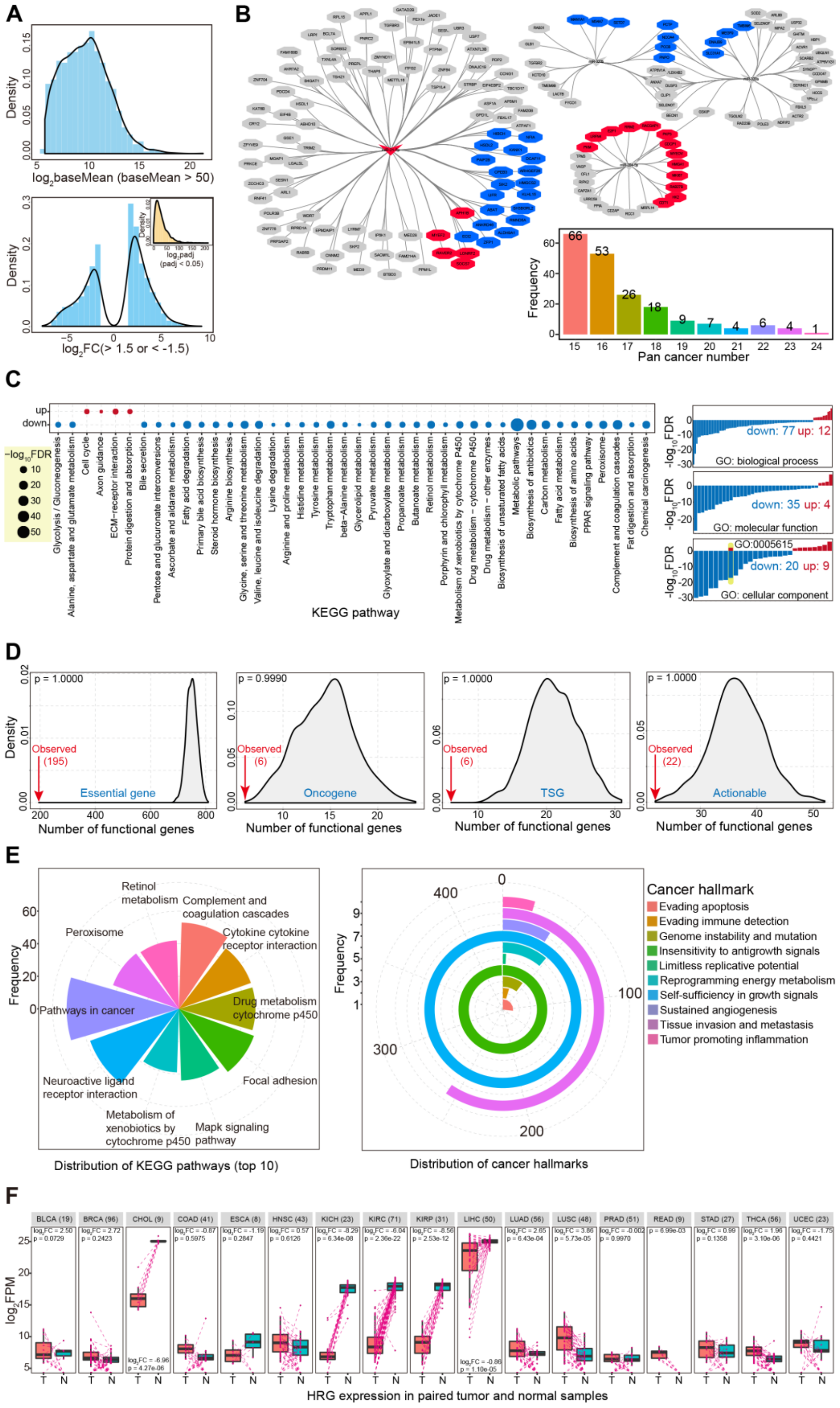

Supplementary Figure 1. Expression patterns of abnormally expressed genes. (**A**) Distributions of baseMean, log_2FC and padj in abnormally expressed mRNAs in CCA. (**B**) An example of miRNA-mRNA interaction. Several miRNAs and their target genes are presented, and all of these miRNA-mRNA interactions are detected at least in 15 cancer types (the detailed distributions are presented below the network).

All of these involved mRNAs are dominantly expressed in CAA. Blue circle shows down-regulated miRNA or mRNA in CAA, red circle shows up-regulated miRNA or mRNA, and grey circle shows normally expressed miRNA and mRNA. (**C**) Enriched significant KEGG pathways of up-regulated and down-regulated mRNAs, and enriched significant GO terms are shown on the right. (**D**) Random testing for distributions of functional genes based on normal genes. (**E**) Distributions of KEGG pathways and cancer hallmarks according to abnormally expressed genes. (**F**) HRG expression in paired tumor and normal samples, and the log_2FC value and p value are also presented using paired t-test. T indicates tumor samples, and N indicates paired normal samples. The total sample size is presented after cancer name.

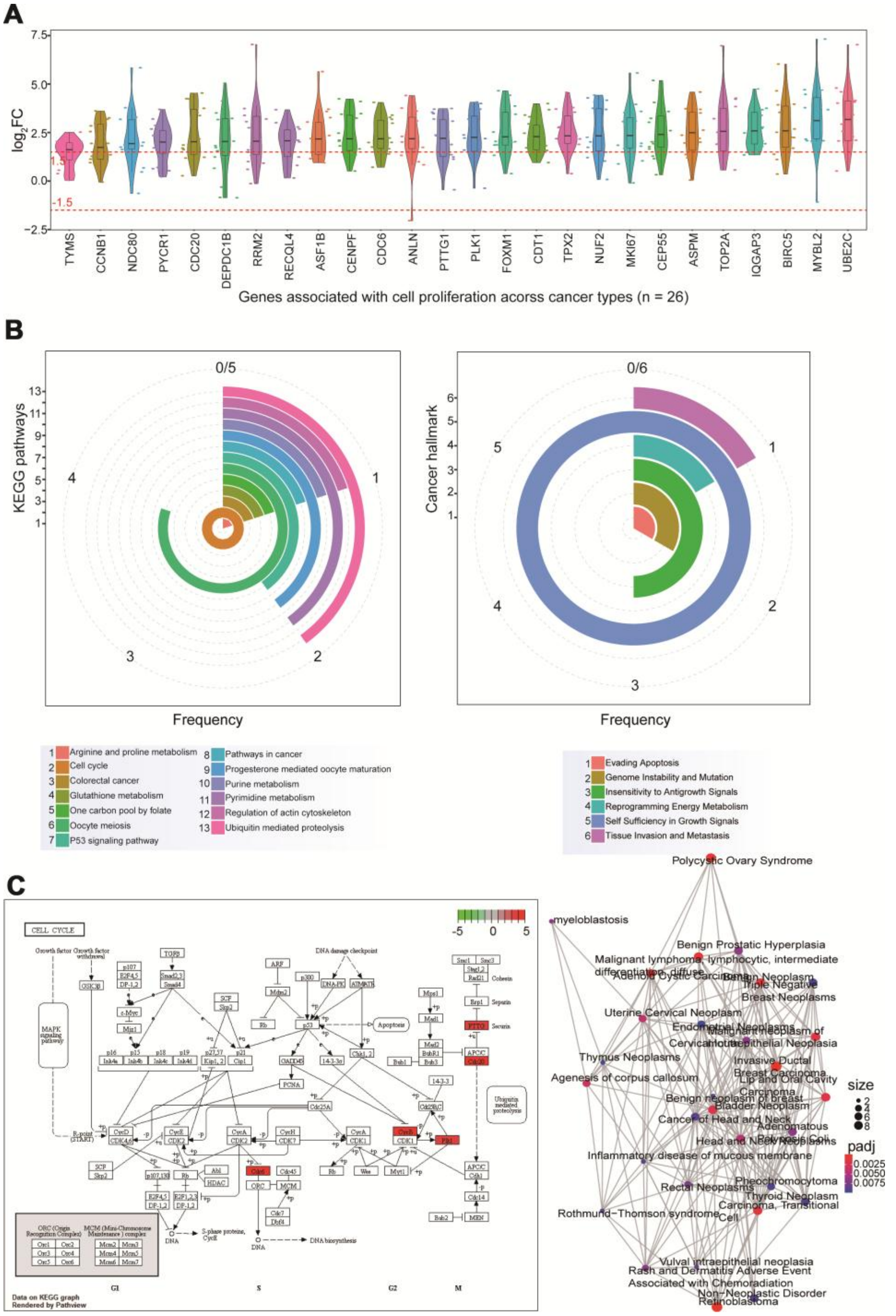

Supplementary Figure 2. Gene distribution of screened genes associated with cell proliferation. (**A**) Distribution of fold change of 26 genes associated with cell proliferation across diverse cancer types. (**B**) Distributions of KEGG pathways and cancer hallmarks for 26 genes. (**C**) Expression patterns of screened 26 genes in cell cycle pathway based on expression profiles in CCA, and enriched network of related diseases.

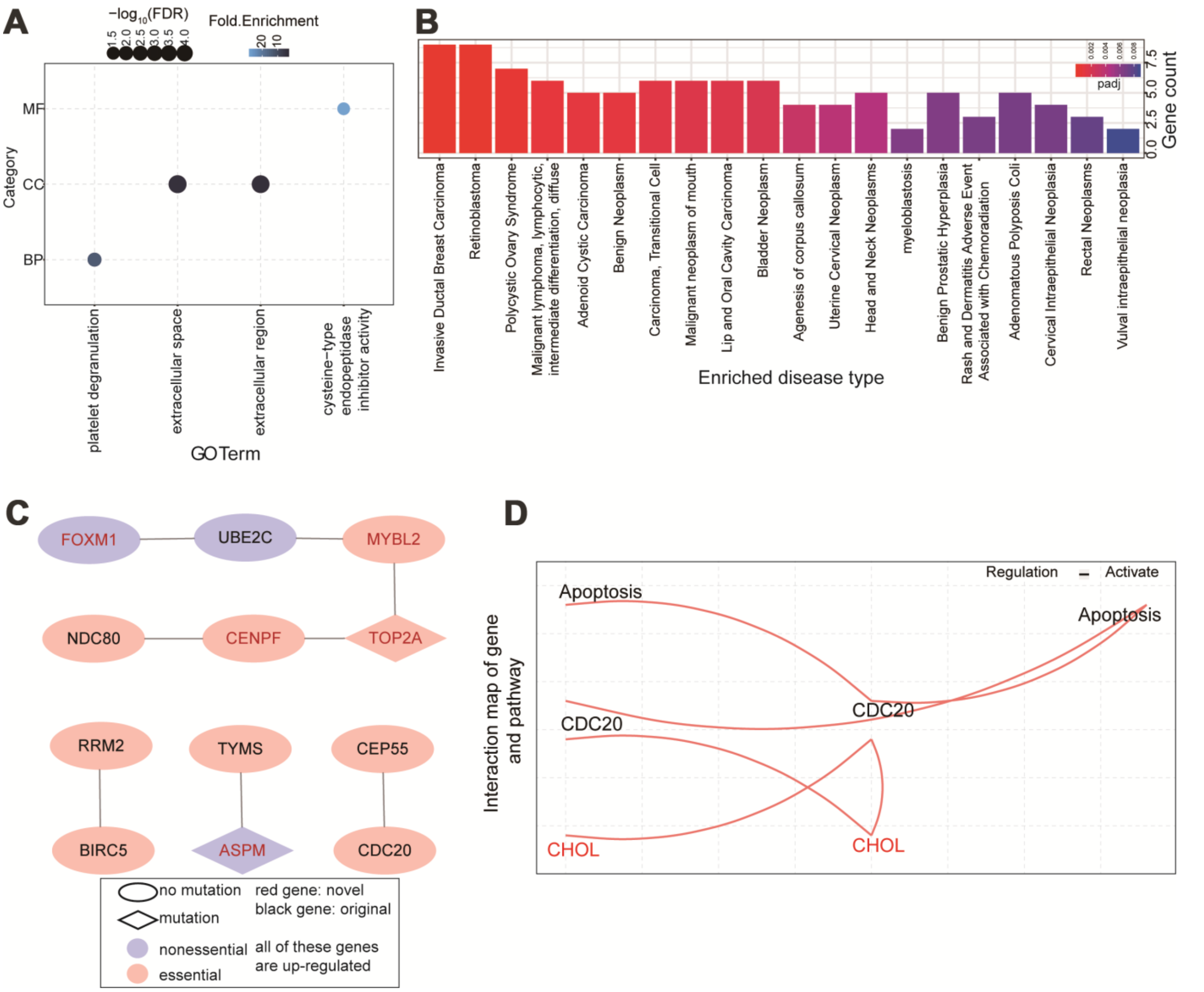

Supplementary Figure 3. Functional analysis and expression distributions. (**A**) Enriched significant GO terms using 94 genes. (**B**) Distributions of enriched diseases based on 26 screened genes associated with cell proliferation. (**C**) Interaction network among 26 screened genes with synthetic lethal genetic interactions. Several gene pairs can be detected among these candidate genes. (**D**) *CDC20* is associated with cell apoptosis.

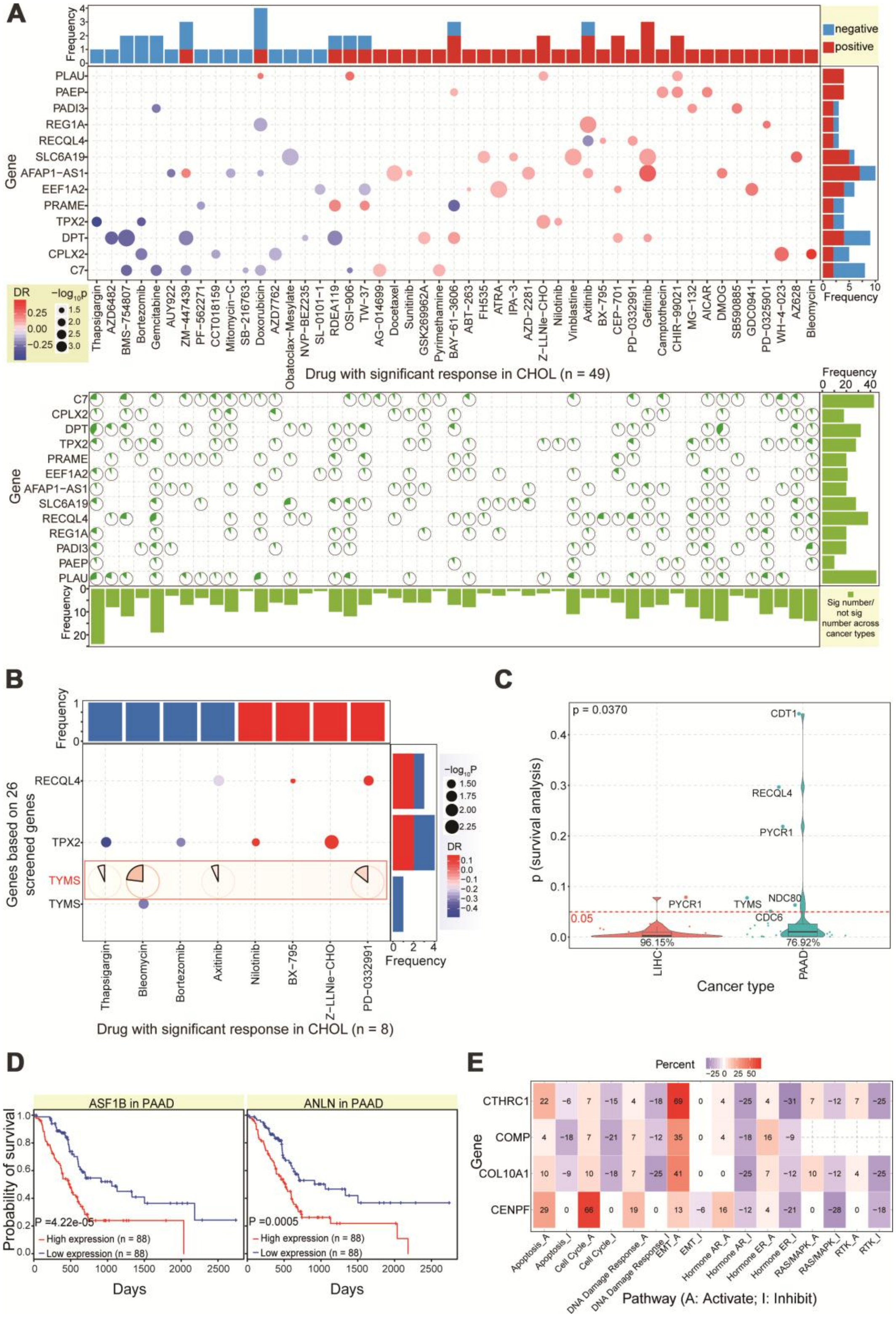

Supplementary Figure 4. Drug response and potential markers for disease prognosis. (**A**) Significant drug response of 55 dominantly expressed genes in CCA (all of these genes have higher enrichment levels in 94 primarily screened genes), and related drug results across cancer types (below figure). This figure only presents significant drug result. (**B**) Only 3 genes associated with cell proliferation are detected significant drug response in CCA. This figure only presents significant drug result. (**C**) Survival analysis of 26 genes associated with cell proliferation in LIHC and PAAD, and most genes show significant correlation with prognosis. No significant difference can be detected between the two cancer types (p = 0.0370). (**D**) Survival analysis of two genes in PAAD, showing their roles in disease prognosis. (**E**) Association of screened 4 genes (their protein levels are deregulated) and biological pathways.
